# A Rare Frameshift Mutation of in *CmACS7* Alters Ethylene Biosynthesis and Determines Fruit Morphology in Melon (*Cucumis melo* L.)

**DOI:** 10.3390/plants14142087

**Published:** 2025-07-08

**Authors:** Jiyang Zhou, Xiaobing Ma, Qianqian Deng, Zhicong Zhong, Xuefei Ning, Li Zhong, Xianliang Zhang, Xianlei Wang

**Affiliations:** 1 Xin Jiang Key Laboratory of Biological Resources and Genetic Engineering, College of Life Science and Technology, Xinjiang University, Urumqi 830000, China; zhoujiyang@xju.edu.cn (J.Z.); 17339907917a@gmail.com (X.M.); 13028331037@163.com (Q.D.); 17796422271@163.com (Z.Z.); ningxuefei@xju.edu.cn (X.N.); zhongli6688@126.com (L.Z.); 2 Institute of Western Agriculture, Chinese Academy of Agricultural Sciences (CAAS), Changji 831100, China

**Keywords:** *Cucumis melo* L, *CmACS7*, frameshift mutation, hormone signaling, fruit shape

## Abstract

Fruit shape diversity in melon is governed by complex genetic networks, with ethylene biosynthesis playing a pivotal yet poorly characterized role. In this study, we identified a rare CmACS7^A57V/frameshift^ double mutant through fine mapping of the *fsq2* locus. Ethylene-mediated ovary growth regulation has been completely lost in the CmACS7^A57V/frameshift^ double mutant, driving a transition from elongated to spherical fruit. Transcriptome analysis was performed to clarify the core role of *CmACS7* in the ethylene signaling pathway. The loss of *CmACS7* function regulates key genes in the ethylene responsive factor, cytokinin signaling pathway, and auxin-related genes, resulting in an imbalance in hormone levels. This imbalance directly affects the coordination of cell proliferation and expansion and ultimately determines the fruit morphology. A genetic diversity analysis of public melon germplasm resources indicated that while the CmACS7^A57V/frameshift^ mutation accounts for only 0.5% of the germplasm, it is strongly correlated with the round fruit phenotype and is important for breeding in Xinjiang. The results of this study suggest that CmACS7^A57V/frameshift^ could be used as a molecular marker to accelerate the breeding of melon varieties with excellent fruit morphology and, at the same time, reveal the coevolutionary significance of this gene in the domestication of Cucurbitaceae crops.

## 1. Introduction

Melon (*Cucumis melo* L.) is a widely cultivated fruit crop known for its diverse varieties and significant economic value [1]. Originating from Africa and Asia, melons have been cultivated for thousands of years and are now grown in various climates around the world [2]. The cultivation of melons is particularly prominent in regions with warm temperatures and ample sunlight, which are conducive to their growth and development. This has given rise to a remarkable diversity in melon fruit morphology. Melon production is not only important for local economies, but also plays a crucial role in global agricultural markets. Fruit shape—a key domestication trait—modulates ecological fitness (via seed dispersal efficiency) and agronomic value (through harvestability and market preference), making it a prime target for evolutionary developmental studies. The shape of melon fruits can vary significantly, ranging from round to elongated forms, and these variations are often associated with specific cultivars and market types [3]. The aesthetic appeal of a fruit shape can affect consumer choices, while certain shapes may be preferred for specific culinary uses. Additionally, fruit shape can impact agricultural practices, including harvesting and packaging. Understanding the genetic regulation of fruit shape is essential for breeders aiming to enhance desirable traits and improve the overall quality of melon varieties.

Genetic mapping and QTL analysis have been instrumental in elucidating the genetic architecture underlying fruit shape in melon. Through the use of recombinant inbred lines (RILs) and near-isogenic lines (NILs), researchers have identified numerous QTLs associated with fruit shape traits [2,4,5]. These studies have revealed that specific QTLs are consistently linked to fruit shape across different genetic backgrounds, suggesting that certain genomic regions are critical for the regulation of this trait. The integration of high-density genetic maps with phenotypic data has allowed for more precise localization of QTLs, facilitating the identification of candidate genes that may be responsible for the observed variations in fruit shape [2,6,7]. This knowledge not only enhances our understanding of the genetic basis of fruit morphology, but also provides valuable insights for the application of marker-assisted selection in melon breeding programs.

Genetic variation is a fundamental driver of the diversity observed in melon fruit shapes [2,8]. This variation arises from both allelic differences at specific loci and the polygenic nature of fruit shape traits [9,10]. The presence of these QTLs indicates that fruit shape is not determined by a single gene but rather by the cumulative effects of several genes, each contributing to the overall phenotype. Furthermore, the genetic background of the melon cultivar can significantly influence the expression of these traits, highlighting the importance of understanding genetic diversity in breeding programs aimed at improving fruit shape [11,12,13].

Research on the genetic regulation of fruit shape in melon has revealed significant insights into the underlying mechanisms. The genetic regulation of fruit shape in melon is influenced by several key genes that play critical roles during the early stages of fruit development [14,15]. Among these, genes associated with ovary development are particularly significant, as they establish the foundational structure of the fruit. Notably, the *a* (andromonoecious) and *g* (gynoecious) genes have been identified as major contributors to flower sex [16,17]. In melon, the fruit shape QTL *fs2.2* co-segregates with the Monoecy (separate male and female flowers on the same plant) gene, *CmACS7*, coding for 1-aminocyclopropane-1 carboxylic acid synthase—the rate-limiting enzyme in ethylene biosynthesis, which represses stamen development in female flowers [16]. In cucumber, *CsACS2*, which is orthologous to *CmACS7*, is associated with elongated vs. round fruit [3,16]. Plants with loss-of-function mutations in *CmACS7* and *CsACS2* showed reduced to no ACS enzymatic activity and developed hermaphrodite flowers and round fruits [3,16]. Moreover, expression of CmWIP1 leads to carpel abortion, resulting in the development of unisexual male flowers. CmWIP1 indirectly represses the expression of the andromonoecious gene, CmACS7, to allow stamen development. CmACS7 and CmWIP1 interact to control the development of male, female, and hermaphrodite flowers in melon, ultimately influencing fruit morphology [17].

Finally, genetic associations between *Cucurbitaceae* sex determination genes and fruit development have been proposed. These genes exhibit pleiotropic effects, influencing not only the sex of the flower but also the morphology of the ovary/fruit, which is crucial for determining the final fruit form [3,18,19]. There are exceptions where modified ethylene perception alter ovary and fruit shape independent of floral sex type [20]. The interaction of these genes with other genetic factors further complicates the inheritance patterns of fruit shape, suggesting that a complex network of regulatory mechanisms is at play. Further investigations have identified multiple QTLs associated with fruit quality traits, including fruit shape, through the analysis of near-isogenic lines and diverse genetic backgrounds. These studies collectively underscore the complexity of genetic interactions that govern fruit morphology in melon.

Comparative studies across cucurbit species have provided valuable insights into the genetic architecture of fruit shape. Research has highlighted the conservation of QTLs related to fruit size and shape among cucurbits, including melon, cucumber, and watermelon [4,20,21]. By analyzing the genetic basis of fruit morphology in these species, researchers have identified homologous genes that play critical roles in regulating fruit traits. For instance, the OVATE family proteins (OFPs) and TONNEAU1 Recruiting Motif proteins have been implicated in the modulation of cell division patterns, influencing the final fruit shape [22,23,24]. In melons, homologous OFP proteins co-localize with QTLs that control fruit shape, suggesting their involvement in shape regulation [25]. Specifically, the melon QTL *fsqs8.1/CmFSI8*—which encodes CmOFP13, a member of the OFP gene family—may share homology with SlOFP20 in tomatoes and is associated with changes in ovary and fruit shapes due to varying expression patterns of CmOFP13 [5,19]. These comparative analyses not only enhance our understanding of the genetic regulation of fruit shape in melon, but also facilitate the identification of candidate genes that may be targeted for crop improvement through breeding programs.

The shapes of vegetables and fruits are outcomes of adaptive evolution and human selection. Recent positive selection has been observed at the andromonoecious allele of the *CmACS7* gene in *Cucumis* melo, possibly driven by selection for resource allocation [26] or the pollen donation hypothesis [27]. These scenarios are particularly relevant for *C. melo*, a cultivated species, where increased fruit production would be subject to positive selection. In Xinjiang, there has been a rising preference for small-sized melons, making them the most favored fruit in the region. Breeding efforts have predominantly focused on developing small-sized melon varieties; newly popular cultivars such as Xizhoumi and Hongxincui are small-sized fruit that result in harboring andromonoecious allele control round fruit. These findings highlight the adaptive evolution of *CmACS7* in response to specific environmental pressures and agricultural practices.

This study aims to explore the impact of the *CmACS7* gene on melon fruit development and to deduce the mechanism by which *CmACS7* affects fruit development. Here, we report the cloning and characterization of a novel melon mutant harboring a truncating mutation in *CmACS7*. This investigation endeavors to elucidate the influence of the *CmACS7* gene on melon fruit development and to establish a comprehensive model illustrating how *CmACS7* modulates fruit developmental processes. Our discoveries illuminate the crucial function of *CmACS7*-mediated ethylene production in orchestrating cellular division and elongation, thereby providing novel mechanistic perspectives on the regulation of fruit morphology in plant systems.

## 2. Materials and Methods

### 2.1. Plant Materials

The utilized melon cultivars were sourced from the Molecular Breeding Laboratory at the National Melon Engineering Technology Research Center. Melon Inbred Line No. 9 (*Cucumis melo* var. ameri) features elongated oval fruits, a trait commonly found in monoecious plants, with an average length of 38 cm and a weight of 4.12 kg. HuangPi (*Cucumis melo* var. ameri) produces more spherical fruits, characteristic of andromonoecious plants, with an average length of 24 cm and a weight of 2.44 kg.

In March 2020, a 425-plant population of BC_1_F_1_ progeny resulting from a cross between No. 9 and HuangPi melon cultivars was utilized to investigate the genetic loci influencing melon fruit elongation. During the autumn of 2021, a total of 1245 BC_1_F_3_-derived individuals were cultivated to facilitate the identification of recombinants. In 2023, a comprehensive genetic analysis was conducted by establishing BC_1_F_6_ populations from 51 unique families in Changji (Xinjiang province), leading to the precise delineation of *QTL-fsq2*, a quantitative trait locus governing melon fruit morphology and mass. This systematic approach allowed for a thorough genetic investigation across multiple generations, resulting in the discovery of novel genes and providing valuable insights into the genetic basis of melon fruit traits.

### 2.2. Genomic Information About 1825 Melon Germplasm Resources

Our research consortium has curated a comprehensive genomic database consisting of 1825 melon accessions using next-generation sequencing, resulting in 9.28 terabase pairs of data. This dataset comprises 790 thin-skinned, 971 thick-skinned, 83 wild, and 27 unclassified melon accessions, providing a diverse range of germplasm resources, such as wild species, landraces, and other varieties. The sequence data associated with this study are publicly available under the project accession numbers PRJNA726743, PRJNA335011, PRJEB37978, PRJNA565356, PRJNA529037, and PRJNA565104. Subsequently, 1385 accessions were chosen for genotype and genetic diversity analyses.

### 2.3. Protocols for Phenotypic Characterization of Fruit

Each family had a minimum of five plants, with one fruit allocated per plant. Post-germination monitoring was conducted for 45 days, focusing on individual fruits retained at nodes 13–15. Fruit characterization included the morphometric assessment of dimensional and gravimetric parameters, such as fruit length, width, fruit shape index (length/width ratio), and weight. Sexual differentiation was based on the presence of staminal structures in pistillate flowers, categorizing them as hermaphroditic, while flowers lacking androecial elements were classified as female.

### 2.4. Development and Implementation of Molecular Markers for Fine-Mapping

Twelve polymorphic SSR markers (Table 1) were designed using Primer Premier 5 and anchored to the reference genome melon 4.0 [28]. The expanded mapping population underwent molecular characterization utilizing these validated markers. Progeny were isolated from individual melon plants for genomic analysis. DNA extraction was carried out using a modified CTAB method [29], followed by high-resolution PAGE analysis to detect parental polymorphisms, facilitating precise genetic differentiation of segregating populations. Subsequently, genotypic and phenotypic datasets were integrated for enhanced QTL analysis using IciMapping Version 4.2.

### 2.5. Genomic DNA and Total RNA Sequencing

#### 2.5.1. Library Preparation and DNA Sequencing

Fresh young leaves were collected from each individual and immediately frozen in liquid nitrogen. DNA was extracted using the CTAB method [29]. A total amount of 0.2 μg of DNA per sample was used as input material for the DNA library preparations. Briefly, each genomic DNA sample was fragmented via sonication to a size of 350 bp. The DNA libraries were sequenced on an Illumina platform, and 150 bp paired-end reads were generated. The sequencing data were mapped to the reference genome using the Burrows-Wheeler Aligner (BWA 0.7.17) software [30] to obtain the original mapping results stored in BAM format (parameter: mem -t 4 -R). Then, duplicates were removed from the results using SAMtools (parameter: rmdup). The raw SNP/InDel sets were called using bcftools with the parameter ‘-mv’.

#### 2.5.2. Library Preparation for Transcriptome Sequencing

For transcriptome sequencing, three fresh ovary samples were collected from each experimental group, measuring 6 mm and 12 mm in diameter of ovary for individuals No. 9 and HuangPi, respectively. These samples were immediately frozen in liquid nitrogen. Total RNA was used as input material for the RNA sample preparations, with 2 ng of total RNA for each cDNA library preparation, using the SMARTer Ultra Low RNA Kit for Illumina Sequencing from Clontech according to the manufacturer’s instructions. Libraries were sequenced on the Illumina HiSeq2000 platform, and 30 to 50 million paired-end reads per sample were obtained. Paired-end clean reads were aligned to the reference genome using Hisat2 v2.0.5. The mapped reads were assigned to genes with featureCount (v1.5.0-p3). DESeq2 (version 1.20.0) was used to identify differentially expressed (DE) genes (adjusted *p* value < 0.05 and log2(FC) > 1 or <−1).

To further elucidate the genetic and transcriptomic landscape, leaves and young ovaries in various developmental stages from No. 9 melon and HuangPi melon were sequenced by the Nuohe Zhiyuan Company. We employed TBtools (version 1.098769) and IGV (version 2.11.9) to analyze sequence variations and differential gene expression patterns (DEGs).

### 2.6. Prediction and Analysis of Candidate Genes

Genomic analyses were performed within a defined mapping interval controlling fruit architecture. Candidate genes were pinpointed utilizing CM4.0 transcript annotations [28].

### 2.7. Cloning of CmACS7 in No. 9 and HuangPi

The 6.7 kb *CmACS7* fragments, comprising genomic DNA sequences from No. 9 and HuangPi, were PCR-amplified using primers designed according to the CM4.0 sequence. The amplified product was then inserted into the pEASY^®^-Blunt Zero Cloning Kit (TransGen, Beijing, China) for sequencing. The PCR primer design aimed to achieve a minimum overlap of 300 bp between adjacent fragments.

### 2.8. Genetic Diversity Assessment

Genetic variation analysis of the *CmACS7* coding sequence was conducted on diverse melon accessions. Interpopulation differentiation (FST) was calculated using SAMtools [31].

### 2.9. Data Analysis

Microsoft Excel and IBM-SPSS (25.0) software were used to perform statistical analyses of phenotypes. The results include the mean, standard deviation, range, frequency distribution, and Pearson correlation.

ImageJ2 software was employed to analyze slices, yielding cell size parameters and cell shape parameters. The size parameters included area, circumference, length (maximum Feret diameter), and width (minimum Feret diameter). The shape parameters comprised aspect ratio and roundness, with roundness calculated and expressed as circumference/2 × π × area. Values approaching 1 indicate increased cell roundness [32].

## 3. Results

### 3.1. Morphological Differences in Ovaries and Fruits in Melon

**Figure 1:**
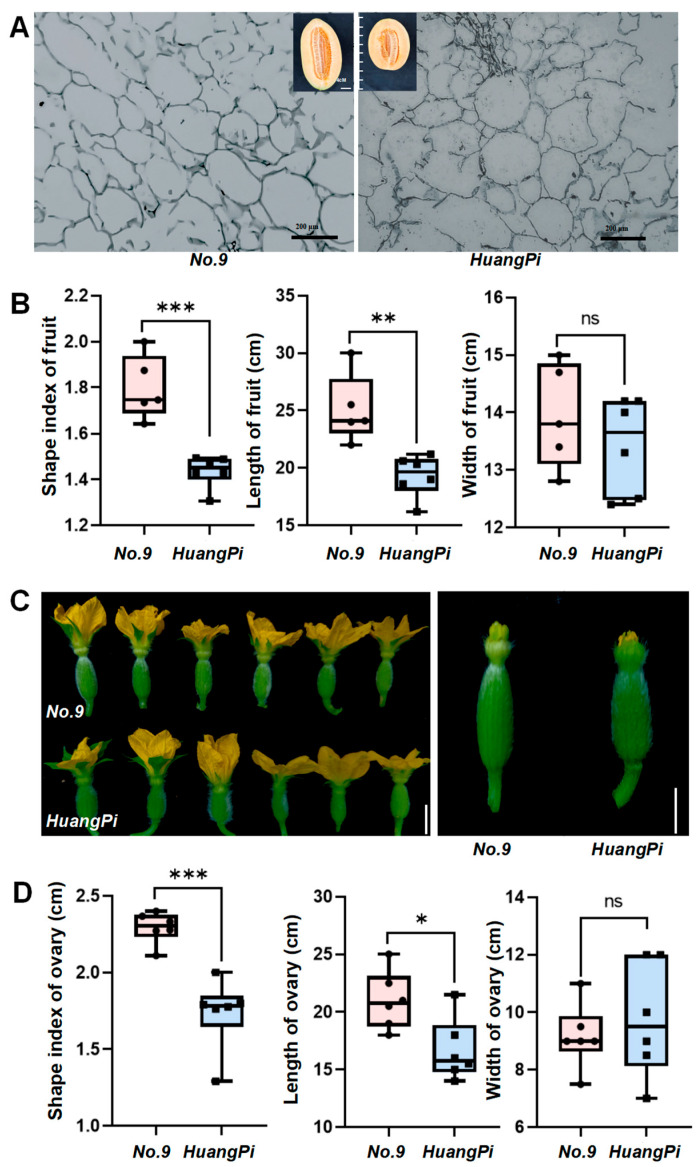
Morphological differences in ovaries and fruits between No. 9 and HuangPi. (**A**) Longitudinal axis paraffin sections of mature fruits from No. 9 and HuangPi. Scale bars, 200 μm. (**B**) Comparison of fruit shape index, length, and width between No. 9 and HuangPi. (**C**) Flower and ovary phenotypes of No. 9 (top) and HuangPi (bottom). (**D**) Comparison of ovary shape index, length, and width between No. 9 and HuangPi. Note: asterisks indicate significant differences (* *p* < 0.05; ** *p* < 0.01; *** *p* < 0.001; ns, not significant; two-tailed Student’s *t*-test). Values are mean ± SD derived from six fresh fruits or ovaries.

In this study, we examined the changes in morphology through developmental stages from ovary to fruit for No. 9 and HuangPi, respectively. First, the length, width, and fruit shape index of the ovaries and fruits were measured and statistically analyzed. Considerable differences were found in flowers and fruit shape between No. 9 (monoecious, longer ovaries and fruits, larger fruit shape index, and greater weight) and HuangPi (andromonoecious, shorter ovaries, and round fruits) (Figure 1A–D). To further investigate the cell numbers and lengths in mature fruits, we compared the fruit cell characteristics in female and hermaphrodite flowers. Our observations revealed that the female fruit had more cells with smaller sized cells along the longitudinal axis than the hermaphrodite fruit (Table 2). Interestingly, despite these variations, the width of both the ovaries and fruits remained consistent, with no significant differences observed (Figure 1B,D).

**Figure 2:**
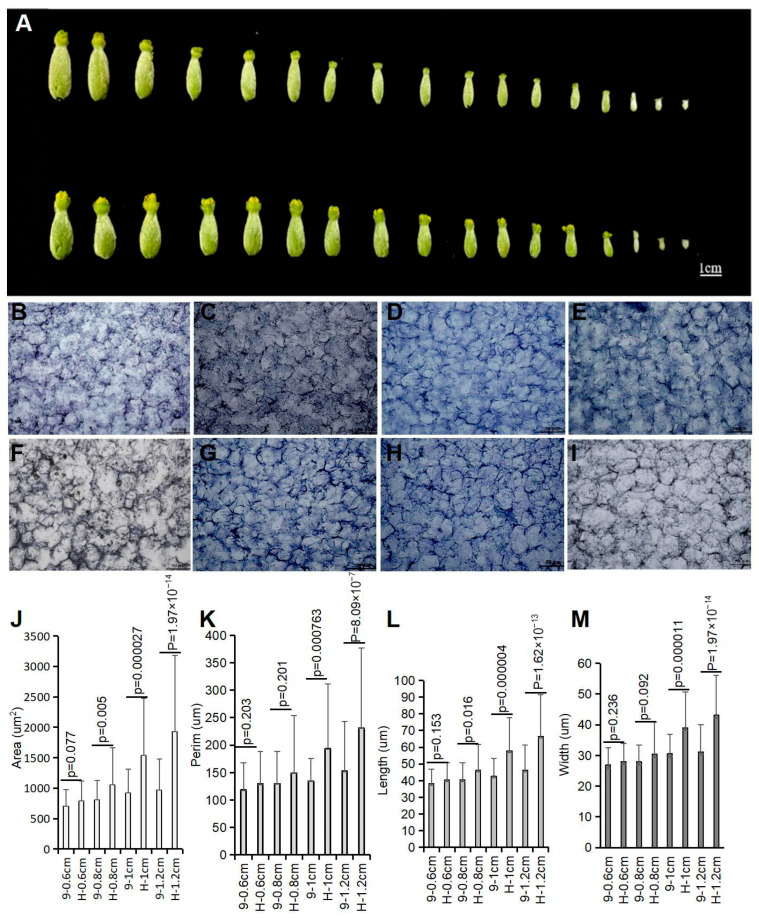
The morphological development of ovaries in No. 9 and HuangPi. (**A**) Changes in ovary size during development. Note: The upper layer represents No. 9, and the lower layer represents HuangPi. Ovarian lengths from left to right are as follows: 2.1, 2.0, 1.9, 1.8, 1.7, 1.6, 1.5, 1.4, 1.3, 1.2, 1.1, 1.0, 0.8, 0.6, 0.5, 0.4, and 0.3 cm. (**B**–**I**) Magnified longitudinal sections of ovaries in different development stages in No. 9 (**B**–**E**) and HuangPi (**F**–**I**). (**J**–**M**) Statistics of cell morphology of ovaries in No. 9 and HuangPi. Frozen sections of ovaries with lengths of 0.6, 0.8, 1, and 1.2 cm. Scale bar: 50 μm. Statistical analysis was performed using a two-tailed Student’s *t*-test. Data represent mean ± standard deviation from six ovarian samples.

Furthermore, ovaries from No. 9 and HuangPi, representing different developmental stages, were meticulously collected and documented (as shown in Figure 2A). We found a notable distinction after the 0.8 cm ovary stage; ovaries from No. 9 were slender, in contrast to the round observed in HuangPi. We measured the cell area, perimeter (perim), length, and width along the longitudinal axis of female and hermaphrodite flowers at various developmental stages, as shown in Figure 2. We found that the cell area, perimeter, length, and width were significantly larger in hermaphrodite flowers (Figure 2J–M). Conversely, female flowers exhibited a significantly higher number of cells with smaller lengths and widths.

A significant positive correlation was found between the morphological structure of the ovary and the mature fruit. This implies that the differences in the initial cell characteristics between female and hermaphrodite flowers play a role in the fruit development process.

### 3.2. Universality of the Major QTL Controlling Fruit Shape in C. melo

**Figure 3:**
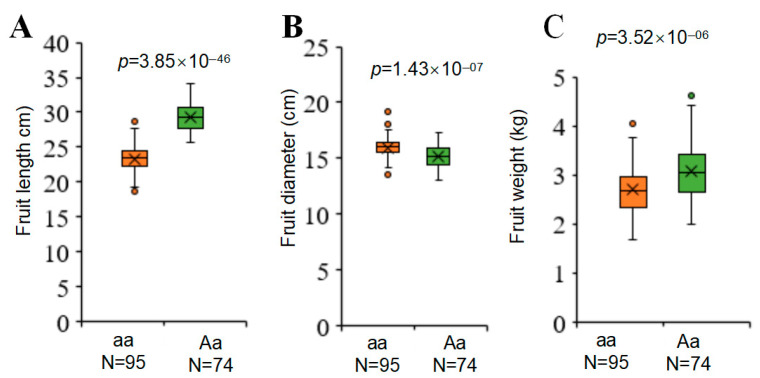
Comparison of the fruit length, width, and weight of plants in the BC1F1 population. (**A**) Comparison of the fruit length of plants in the BC1F1 population. (**B**) Comparison of the fruit weight of plants in the BC1F1 population. (**C**) Comparison of the fruit width of plants in BC1F1. Statistical analysis was performed using a two-tailed Student’s *t*-test. Data represent mean ± standard deviation from samples carrying aa and Aa genotypes.

To determine whether the andromonoecy locus determines fruit shape in No. 9 and HuangPi, we constructed a 425-plant BC_1_F_1_ population by crossing No. 9 with HuangPi (background). The phenotypic ratio between monoecious and andromonoecious plants was close to 1:1 (x^2^ = 1.25, df = 1, *p* = 0.2646), indicating that this floral trait is controlled by a dominant gene, possibly the effect of the andromonoecy locus [3,16]. We developed a genetic marker for *CmACS7* to assess sex differentiation in the BC_1_F_1_ population based on previous findings that the *CmACS7* gene controls floral traits [3,16]. We found that sex traits co-segregated with *CmACS7* in the BC_1_F_1_ population (Table S1). At the time of harvest, we randomly selected 169 plants from BC_1_F_1_ and measured the length, width, and weight of the fruits. *CmACS7* significantly influenced the fruit shape and weight (Figure 3A–C). These findings suggest that variations in floral traits and fruit shape may be attributed to the pleiotropic effects of the andromonoecy locus. Consequently, this locus was designated *fsq2*, *flq.2*, or *fwq.2* in a previous study [4].

**Figure 4:**
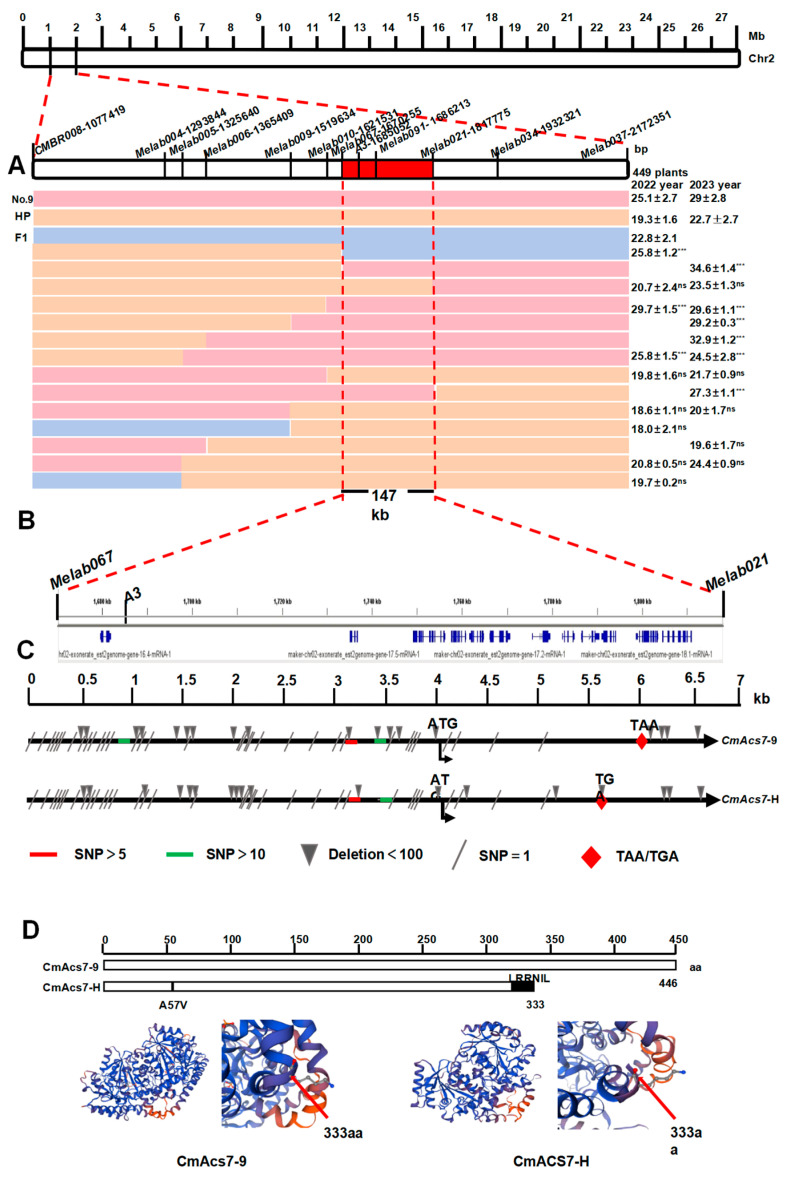
Mapping and cloning of *fsq2*. (**A**) Fine-mapping of *fsq2*. (**B**) Candidate genes within *fsq2*. (**C**) Sequence alignment of *CmACS7* with different accessions. (**D**) Comparison of protein modules of CmACS7 in different accessions; CmAcs7-9 represents CmAcs7 in No. 9 and CmAcs7-H represents CmAcs7 in HuangPi. Statistical analysis was performed using a two-tailed Student’s *t*-test. Data represent mean ± standard deviation from six mature fruits length. *** Indicates significant correlation at 0.001 level; ns means no difference. All data compared with HuangPi (HP) phenotype.

To confirm the pleiotropic effects of *CmACS7*, we developed 11 SSR and 1 CAPs polymorphic markers in a 1 Mb genomic region linked to *CmACS7* for genetic mapping (Figure 4A, Table 1). A total of 1052 plants from the BC_1_F_4_ population were subjected to genotyping using PCR. We subsequently incorporated polymorphic markers to genotype 151 recombinant plants. Fruit shape data from the years 2022 and 2023 were used to identify the *fsq2* gene, which was found to be flanked by the genetic markers *Melab067* and *Melab021*. The gene was estimated to be located at a physical distance of 147 kilobases. To improve annotation, we consulted the melon genome database available at the Cucurbit Genomics Database website. The database can be accessed at http://cucurbitgenomics.org/organism/18, accessed on 2 July 2025. It is hypothesized that there are 12 annotated genes within the localization interval based on the provided annotation data. This information is depicted in Figure 4B and Table 3.

#### 3.2.1. Identification of Sequence Variations in the fsq2 Candidate Interval

**Figure 5:**
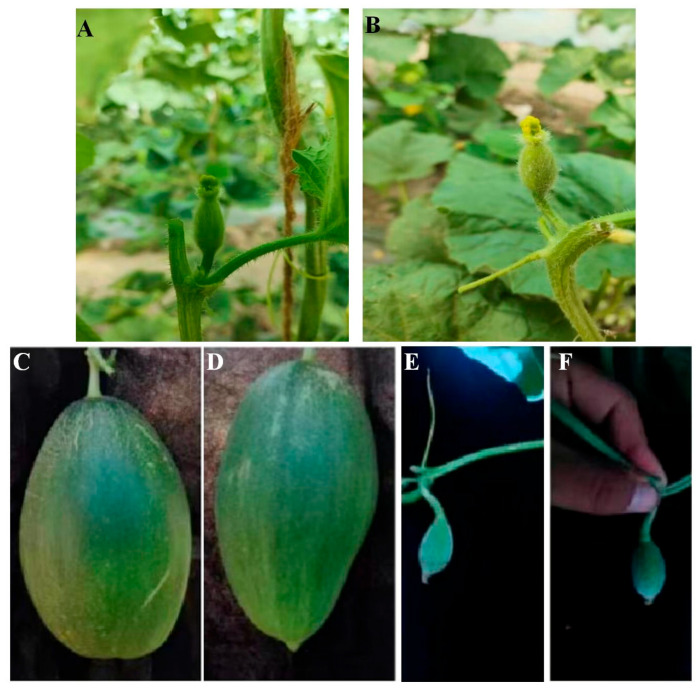
Silver nitrate treated No. 9 plants develop hermaphrodite flowers and fruit. (**A**,**B**) correspond to representative female and hermaphrodite flowers from a total of 6 hermaphrodite flowers from 12 independent nontreated and treated plants. (**C**–**F**) correspond to representative fruit shape from 12 independent nontreated and treated plants.

We conducted re-sequencing analysis of parental No. 9 and HuangPi identified sequence variations within the candidate interval of *fsq2*. Among the coding sequence (CDS) regions of the 12 genes, eight variations were detected, comprising four synonymous mutations, three missense mutations, and one frameshift mutation (Table 4). The mutations were identified in the genes *MELO3C015444*, *MELO3C015454*, and *MELO3C015455*, as detailed in Table 4. The gene *MELO3C015444*, also referred to as *CmACS7*, encodes the enzyme 1-aminocyclopropane-1-carboxylate synthase and has been associated with sex determination in plants. Additionally, PCR cloning of the 6.7 kb *CmACS7* region from both No. 9 and HuangPi revealed two distinct mutations. One of the identified mutations was a missense mutation leading to an A57V amino acid substitution, in line with prior studies. Moreover, frameshift mutations and the premature termination of protein translation were detected due to a single T-base deletion in the coding sequence (CDS) region. These mutations were also identified through PCR cloning of the 6.7 kb *CmACS7* region from both No. 9 and HuangPi. The CmACS7-H protein variant is 113 amino acids shorter than CmACS7-9, resulting in notable structural disparities in the anticipated protein configuration (Figure 4D). This difference in length between the two protein variants has implications for their overall structure and function. Structural modeling predicts that the A57V/frameshift mutation disrupts the ACS7 catalytic domain (α-helix deletion, Figure 4D). We know that CmACS7, encoding 1-aminocyclopropane-1 carboxylic acid synthase, is the rate-limiting enzyme in ethylene biosynthesis. Ethylene is likely a positive activator of elongated fruit growth [3]. To test this hypothesis, we treated No. 9 melon plants with 1200 ppm of the ethylene perception inhibitor, silver nitrate. As expected, treated plants developed hermaphrodite flowers (Figure 5). In summary, the data robustly support the involvement of ethylene synthesized in the flower through monoecy genes in the elongated fruit development process.

#### 3.2.2. Molecular Insights into Ovary Shape Regulation

**Figure 6:**
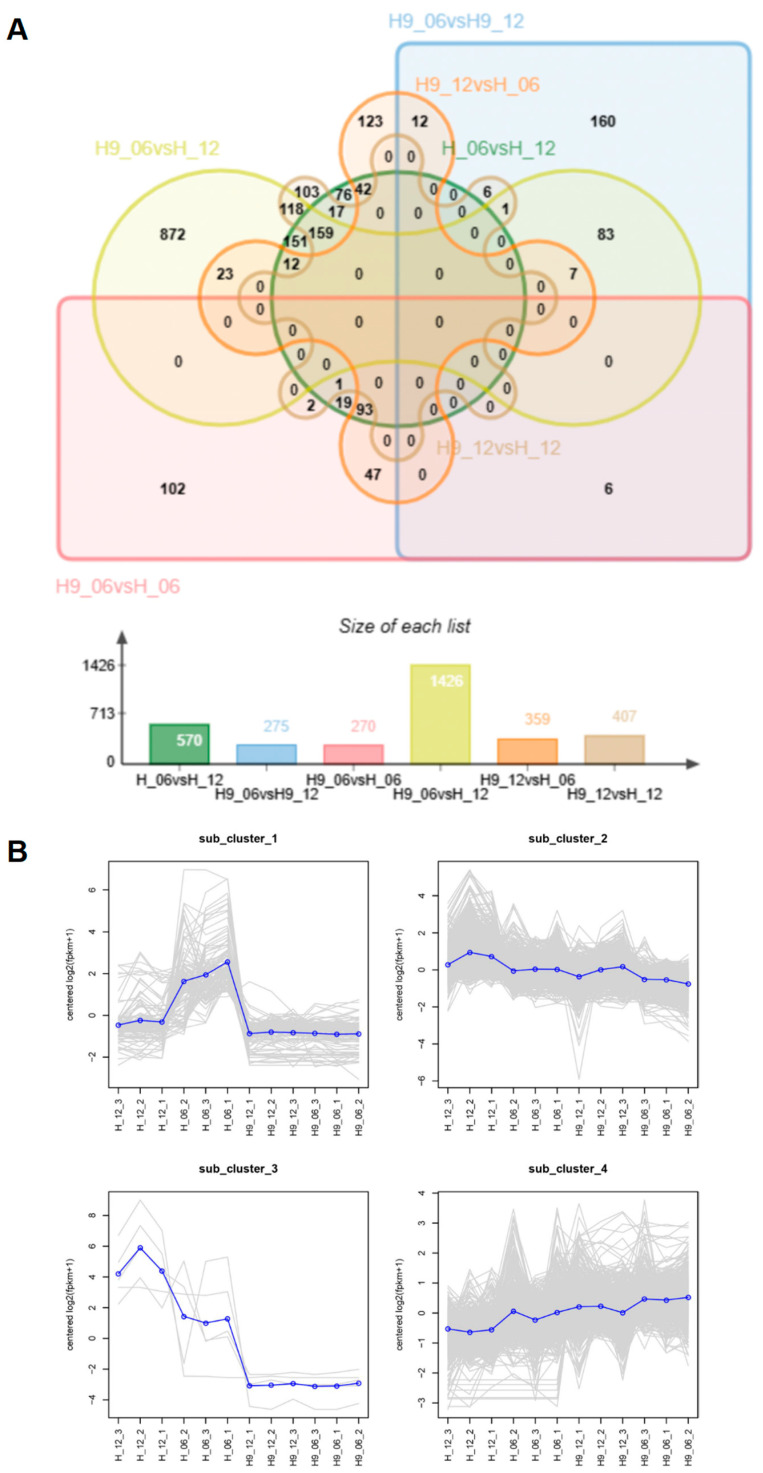
Gene expression profiling of female and hermaphrodite flowers by RNA-seq. (**A**) Venn diagram showing overlap between pairwise comparison groups [33]. (**B**) Analysis of expression patterns of differentially expressed genes. Number of differentially expressed genes in each cluster: N = 99, N = 1606, N = 5, N = 855.

**Figure 7:**
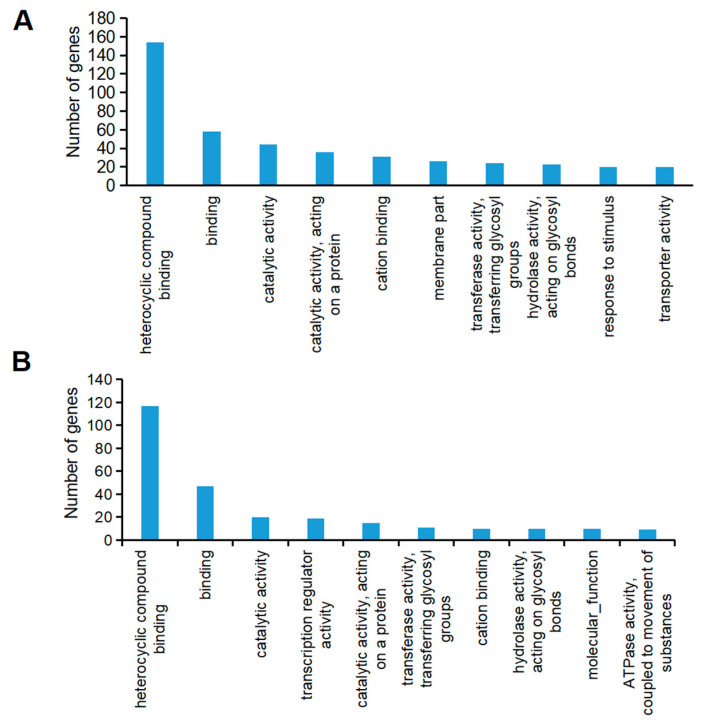
Top 10 enriched GO terms in clusters 2 and 4 are shown in (**A**,**B**).

The shape of the ovary is intricately influenced by two pivotal developmental processes: cell division and cell elongation. These processes play significant roles in determining the overall structure and function of the ovary. To gain a nuanced understanding of the molecular mechanisms that modulate these processes and ovary shape, the transcriptome sequencing was performed on ovaries at the 0.6 cm and 1.2 cm developmental stages for female and hermaphrodite flowers. Pairwise comparisons revealed that hermaphrodite flowers at the 1.2 cm stage had the greatest number of uniquely expressed genes, as depicted in Figure 6A. In total, 2565 differentially expressed genes were discovered across various stages of ovary development, as shown in Figure 6A. These genes were categorized into four distinct clusters based on their expression patterns, as depicted in Figure 6B. GO term enrichment analysis revealed that clusters 2 and 4 were particularly enriched with terms related to heterocyclic compound binding, various binding activities, catalytic functions, actions on proteins, cation binding, membrane components, transferase activities, and hydrolase activities. Notably, cluster 2 was associated with response to stimulus and transporter activities, while cluster 4 was linked to transcription regulator activity, molecular function, and ATPase activity, as detailed in Figure 7A,B.

**Figure 8:**
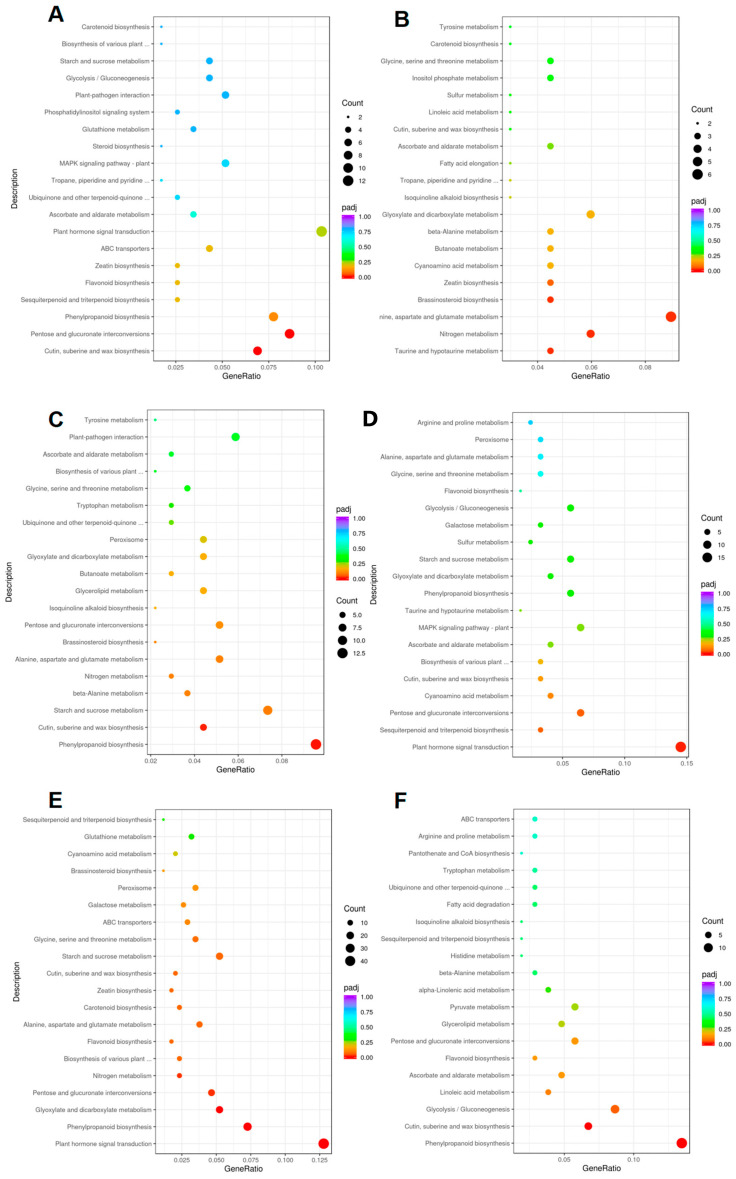
KEGG analysis of differently expressed genes. (**A**) Genes in 0.6 cm and 1.2 cm ovaries in HuangPi. (**B**) Genes in 0.6 cm and 1.2 cm ovaries in No. 9. (**C**) Genes in 0.6 cm ovaries in HuangPi and No. 9. (**D**) Genes in 1.2 cm ovaries in HuangPi and No. 9. (**E**) Genes in 1.2 cm ovary in HuangPi and 0.6 cm ovary in No. 9. (**F**) Genes in 0.6 cm ovary in HuangPi and 1.2 cm ovary in No. 9.

In-depth analysis of the KEGG pathways associated with differentially expressed genes revealed significant differences in the developmental mechanisms between HuangPi and No. 9 ovaries. The development of HuangPi ovaries appears to be primarily driven by plant hormone signal transduction pathways, as presented in Figure 8A. This suggests that hormonal cues are pivotal in shaping the maturation process of HuangPi ovaries. Conversely, the developmental trajectory of No. 9 ovaries is strongly linked to the biosynthesis of alanine, aspartate, and glutamate, as shown in Figure 8B. These amino acids are fundamental to protein synthesis and cellular metabolism, indicating their critical role in the growth and ripening of No. 9 ovaries. To gain a more comprehensive understanding of the relationship between ovary development and fruit shape, we extended our analysis to include the developmental processes of various ovary types. We discovered that the key factors influencing ovary development are largely involved in plant hormone signal transduction pathways and phenylpropanoid metabolism pathways, as illustrated in Figure 8C,F.

**Figure 9:**
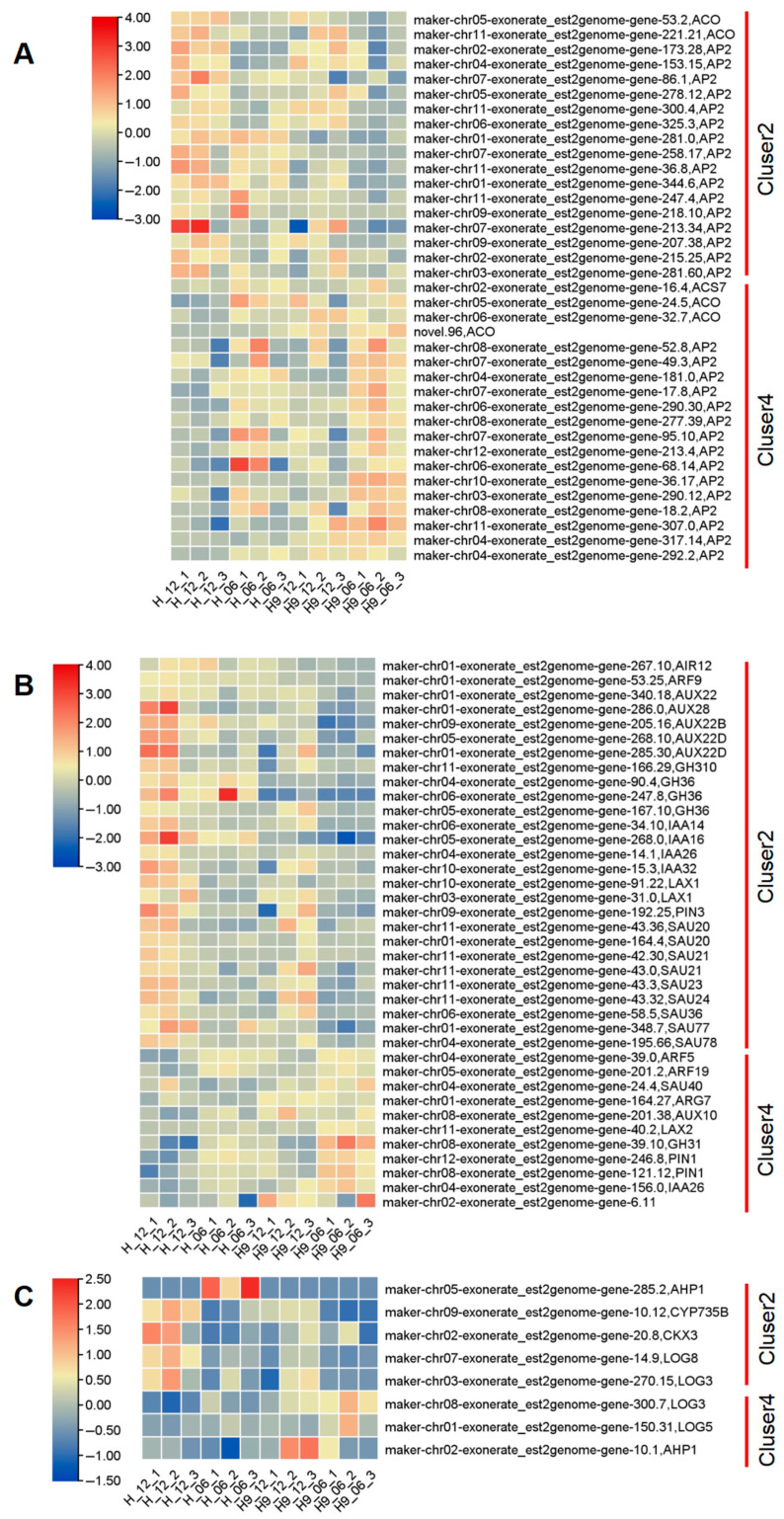
RNA-seq heat maps of differentially expressed genes related to plant hormone signal transduction in different ovaries. (**A**) Ethylene responsive factor. (**B**) Auxin-related genes. (**C**) Cytokinin-related genes.

Genomic analysis revealed that *CmACS7-H* is a loss-of-function mutation associated with changes in ovary morphology. This genetic variant was found to be responsible for the observed variations in the ovary phenotype. Aiming to understand the underlying phenotypic variation, we found that the expression level of *CmACS7* decreased significantly during ovary development (Figure 9A). The expression levels of CmACS7 were not detected in other tissues, such as root, stem, leaf, and flower, by semi-RT PCR. The results suggested low expression levels observed in other tissues. Therefore, we focused on genes implicated in plant hormone transduction pathways, which are known to influence melon fruit shape. An examination of differentially expressed genes showed that more than 50% of the genes related to ethylene production and the ethylene response factor (ERF family) were activated in the H12 sample compared to the 9-06, 9-12, and H06 samples (Figure 9A). Furthermore, more than two-thirds of the genes related to auxin biosynthesis and the auxin response factor (ARF) were also upregulated in H12, as shown in in Figure 9B. This finding suggests a potential regulatory role of auxin-related genes in the biological processes associated with H12. The results are consistent with the decrease in cell count and increase in cell size across the width of the ovary in the H12 sample compared to the 9-06, 9-12, and H06 ovaries. Furthermore, gene expression associated with Zeatin, an important phytohormone, exhibited variation during ovary development in distinct samples (Figure 9C). The results highlight the significance of *CmACS7* expression and its relationship with ethylene and auxin signaling in influencing the morphology of melon ovaries and fruits. This indicates that there is developmental-specific coordination between *CmACS7* and hormonal pathways during early stages of growth of melon fruit.

#### 3.2.3. CmACS-H May Play a Vital Role in Melon Breeding

To further elucidate the genetic diversity of the *CmACS7* gene among melon germplasm resources, we conducted a comprehensive analysis of coding sequence (CDS) variations across 1385 melon accessions. Our findings revealed a total of 28 mutations within the *CmACS7* coding region (Table 5). A C-T transition at the 170th base results in A57V (chr02:1679936), which not only changes the floral trait from monoecious to andromonoecious but also changes longer fruit to round fruit, according to a previous study [3,16]. The highest nucleotide diversity (π) observed in the entire population at chr02:1679936 suggests significant nucleotide variation among individuals (Table 5). Furthermore, the presence of a *CmACS7* frameshift at position chr02:1681415 was associated with a lower Fst value and reduced nucleotide diversity, indicating limited genetic variation among the studied individuals. Further investigation revealed that this unique allelic variation is specific to nine accessions of *Cucumis melo* L., including Hongxin Crisp, Xizhou Honey 25, 2010Xj6, Smooth Hairless, Empress, Yujin, Bokezade, and Guowei. Interestingly, these accessions are exclusive to the Xinjiang planting region.

## 4. Discussion

Fruit morphology, a quantitative characteristic, is determined by an intricate interaction of numerous genes and complex genetic regulatory pathways. Understanding these mechanisms can enhance fruit quality and yield in agricultural crops. To date, several QTLs associated with fruit shape have been cloned within the Cucurbitaceae family, including *CmACS7* [3,16], *CmCLV3* [18], and *OFP* [5,19], all of which significantly contribute to the variation in fruit shape. The specific molecular mechanisms and regulatory pathways through which these genes influence fruit shape are not yet fully understood, requiring more research efforts to uncover them.

We discovered a new allelic mutation in *CmACS7*, designated as *CmACS7-H*. *CmACS7-H* not only has an A57V mutation but also harbors a frameshift mutation. The natural double mutants were analyzed to understand the effects on plant growth and development. Furthermore, CmACS7 exhibits a significant degree of similarity among angiosperms, particularly with CsACS2, suggesting a shared biological function between CmACS7 and CsACS2. Boualem et al. [3] demonstrated that CsACS2 controls the morphology of cucumber fruit. CsACS2^G33C^, CsACS2^P209S^, and CsACS2^S399L^ isoforms showed reduced to no ACS enzymatic activity and developed hermaphrodite flowers and round fruits. These results suggest that mutations at various loci within CmACS7 can result in alterations in gene functionality. These mutations may affect the gene’s activity in different ways. This parallel discovery suggests that CmACS7^frameshift^ and CsACS2^S399L^ may play the same role in regulating flower development and fruit shape in plants. As the loss of the enzymatic activity of CmACS7/CsACS2 is associated with round fruit development, ethylene is likely a positive activator of elongated fruit growth, explaining its complete loss-of-function phenotype.

In melon, genetic diversity analysis has shown that the *CmACS7* allele leading to andromonoecy is the result of recent positive selection [16], either to permit flexibility in resource allocation to male and female functions or to elevate male function and, thus, increase pollen donation. Notably, the CmACS7^A57V^ allele prevails in melon accessions developing round fruits [3]. Here, we also show that the natural double mutant CmACS7^A57V/frameshift^ entirely eliminates its role in regulating ovary growth. This novel allelic variation was detected in nine accessions, such as Hongxin Crisp, Xizhou Honey 25, 2010Xj6, Smooth Hairless, Empress, Yujin, Bokezade, and Guowei. Despite the low PI value, the existence of these germplasm resources containing the *CmACS7-H* genotype has played a significant role in the development of high-quality melon varieties in the region. These results indicate that the diversity in fruit morphology can be controlled by the dosage impact of *CmACS7*, playing a crucial role in melon breeding in Xinjiang. It highlights the importance of *CmACS7* in the development of melon varieties with desired fruit shapes and sexual characteristics, which have been favored by continuous selection in different regions around the world.

Fruit shape is the result of coordinated cell division and expansion. The plant hormone ethylene plays a key role in flower and fruit development. We found that *CmACS7*-mediated ethylene production affects both the development of elongated fruits and female flower development. RNA-seq analysis has shed light on the distinct developmental pathways involved in ovary maturation in HuangPi and No. 9. Plant signal transduction pathways play a significant role in governing the development of the HuangPi ovary. In contrast, the alanine, aspartic acid, and glutamate synthesis pathways are more crucial for the development of the No. 9 ovary. This divergence underscores the complexity of fruit development, with different genotypes harnessing distinct metabolic and signaling mechanisms.

**Figure 10:**
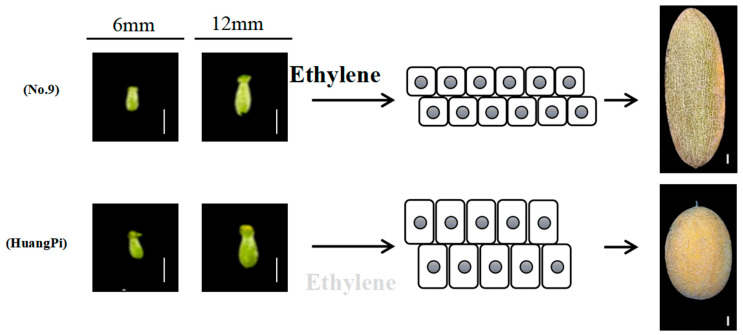
A model of the effect of the CmACS-7 gene on fruit development.

By integrating genetic and transcriptomic data, we propose a mechanistic model where CmACS7 dosage modulates growth polarity (Figure 10). During early ovary development, high expression of the CmACS7-9 gene leads to an increased number of cells per unit area and smaller cell sizes, resulting in elongated fruit. Conversely, the CmACS7-H gene is functionally deficient, potentially nullifying CmACS7′s role in regulating round ovary development.

## 5. Conclusions

This study aimed to investigate the influence of CmACS7 on fruit shape in melon. Further understanding of these complex interactions is essential for unraveling the genetic mechanisms that control fruit development and for informing breeding programs aimed at improving fruit characteristics in melon. We successfully cloned a new double mutant of CmACS7, known as CmACS7^A57V/frameshift^. This mutation may entirely eliminate the role of CmACS7 in controlling round ovary development. Finally, we proposed a model to address the relationship between CmACS7 expression, hormonal signaling, and cellular growth patterns, which together dictate the final morphology of the fruit. While our genetic analysis confirmed CmACS7′s necessity, the spatiotemporal dynamics of its protein interactions remain uncharacterized. In addition, the conserved ethylene biosynthesis pathway suggests the translational potential of these results for CmACS7 homologs in Cucurbitaceae, where similar mutations could promote compact fruit size—a trait demanded by urban agriculture and premium markets. Targeted editing of CmACS7 homologs offers a CRISPR-compatible route to develop mini-fruit cultivars in Cucurbitaceae, addressing space-efficient cultivation needs.

## Figures and Tables

**Table 1:** List of primer sequences.

Primer	F	R
CMBR008	TTTCACTTTTTCCCGCCG	AATGGAAAAGGGAAGTGCAA
Melab004	CGGACTTATTATTCCCACAA	CGTTTCTTCGAGGTGTTA
Melab005	ATCAGGCATGAAGCGGAAA	CGACCAACCATTGCTCACTC
Melab006	AATTTTGATTTAGGCTTGG	ATAAACCAAACATCCCCAC
Melab009	GACATAGCCATGATGTTGAG	TTCTTCTATACCCATCTTTTC
Melab010	ATAAACATAGGACACCGAGAA	GGATTGATTTTACCGCACA
A3	ATCTCAACATCTACCAAA	CCTCCTATTACATTTTC
Melab021	CTCTTTATTACCCACTTCTC	TCTAATGTTTGAGCAAGTCC
Melab034	GGGAAGTCCTACGCATCATA	TAAGTTTGGGGTGGTGAGCA
Melab037	ACCATAAATCTTGCCAAAATA	TTGACCGTAAGTTCTTGTTGG
Melab067	GATAATCAAATCCTTAGTAGAA	TCTAAATCTATTCATCGATCAC
Melab091	ACTTGGTCGTTGTAATAAAA	CTCTACTCGTAAACATTGCC
For cloning		
Melab048	CGGGATCCTTGTCCCTAAAATACTCCAT	GGGGTACCAGACAAAGGAAATCAGCAA

**Table 2:** Statistics of cell morphology of mature fruits in No. 9 and HuangPi.

Accessions	Number of Cells per Unit Area	Cell Size				Morphology	
		Single-Cell Area/μm^2^	Perimeter/μm	Length/μm	Width/μm	Aspect Ratio	Roundness
No. 9	30	13,120.08	496.96	158.42	95.75	1.74	0.62
HuangPi	25	21,162.96	667.42	208.31 **	129.64 ***	1.67	0.65

Note: asterisks indicate significant differences (** *p* < 0.01, *** *p* < 0.001, two-tailed Student’s *t*-test). Values are means derived from 10 microplants. Number of cells per unit area means cell number in the field of view under a microscope in longitudinal axis.

**Table 3:** Candidate genes predicted in candidate regions.

GENE	Name	Predicted Gene Function
1	MELO3C015444	1-Aminocyclopropane-1-carboxylate synthase
2	MELO3C015445	Peroxidase
3	MELO3C015446	xaa-Pro dipeptidase
4	MELO3C015447	Protein arginine N-methyltransferase
5	MELO3C015448	AT4G29520-like protein
6	MELO3C015449	DNA ligase 4
7	MELO3C015450	Pollen-specific protein SF21
8	MELO3C015451	Inorganic pyrophosphatase 2-like
9	MELO3C015452	Mitochondrial carrier protein
10	MELO3C015453	Protein EFFECTOR OF TRANSCRIPTION 2
11	MELO3C015454	Inactive protein kinase SELMODRAFT_444075
12	MELO3C015455	Acyl--UDP-N-acetylglucosamine O-acyltransferase

**Table 4:** Variations in the coding regions of genes in the mapping interval.

Chr	Position	Ref	No. 9	HuangPi	Variation	Gene	Ref	No. 9	HuangPi
chr02	1679775	T	C	T	synonymous	*MELO3C015444*	IIe	IIe	IIe
chr02	1679936	T	C	T	missense	*MELO3C015444*	Val	Ala	Val
chr02	1681416	T	T	/	frameshift	*MELO3C015444*			Premature
chr02	1799392	T	T	C	synonymous	*MELO3C015454*	Thr	Thr	Thr
chr02	1801149	A	A	T	missense	*MELO3C015454*	Met	Met	Ieu
chr02	1801547	C	C	A	synonymous	*MELO3C015454*	Gly	Gly	Gly
chr02	1804515	C	T	C	synonymous	*MELO3C015455*	Ieu	Ieu	Ieu
chr02	1805623	T	T	A	missense	*MELO3C015455*	Ieu	Ieu	His

**Table 5:** Genetic diversity analysis of *CmAcs7* coding region in 1385 accessions.

CHR	POS	PI-Wild	PI-Landrace_Agrestis	PI-Cultivar_Agrestis	PI-Landrace_Melon	PI-Cultivar_Melon
chr02	1679889	0.0251552	0.0262685	0.0061633	0.0653953	0.027059
chr02	1679936	0.325083	0.267292	0.131725	0.222248	0.187948
chr02	1680051	0.141256	0	0	0	0
chr02	1680066	0.142928	0	0	0	0.0322935
chr02	1680114	0.141256	0	0	0	0
chr02	1680147	0.141256	0	0	0	0
chr02	1680189	0.141256	0	0	0	0
chr02	1680223	0.120761	0	0	0	0
chr02	1680267	0.141256	0	0	0	0
chr02	1680291	0.141256	0	0	0	0
chr02	1681034	0.141256	0	0	0	0
chr02	1681076	0.141256	0	0	0	0
chr02	1681094	0.142928	0	0	0	0
chr02	1681113	0.142928	0	0	0	0
chr02	1681163	0.141256	0	0	0	0
chr02	1681224	0.141256	0	0	0	0
chr02	1681229	0.293477	0.0129237	0.0183193	0.00667776	0
chr02	1681256	0.151913	0	0	0	0
chr02	1681259	0.12528	0	0	0	0
chr02	1681262	0.146392	0	0	0	0
chr02	1681346	0.142928	0	0	0	0
chr02	1681367	0.14464	0	0	0	0
chr02	1681385	0.142928	0	0	0	0
chr02	1681415	0	0	0	0.0200317	0.0321207
chr02	1681421	0.141256	0	0	0	0
chr02	1681449	0.142928	0	0	0	0
chr02	1681496	0	0.050681	0.00620152	0.0976292	0.0269134
chr02	1681586	0.14464	0	0	0	0

## Data Availability

Data will be made available on request.

## Supplementary Materials

The following supporting information can be downloaded at: https://www.mdpi.com/article/10.3390/plants14142087/s1, Table S1: Sexual phenotypes in B_1_F_1_ population.
